# Lapatinib-induced inhibition of ovarian function is counteracted by the STAT3 pathway both *in vivo* and *in vitro*

**DOI:** 10.3892/or.2021.8074

**Published:** 2021-05-07

**Authors:** Qiuyue Liao, Xue Feng, Xi Li, Ge Chen, Jing Chen, Bin Yang, Kezhen Li, Jihui Ai

Oncol Rep 44: 1127-1135, 2020; DOI: 10.3892/or.2020.7660

Following the publication of the above article, the authors have realized that the same image had been selected to show H&E staining of the ovarian slices in the Control and 10 μM groups in Fig. 4A. After reviewing the original pictures, the authors discovered that the image correctly showing the data for the 48 h, 10 μM group had also been used for the 48 h Control group.

The corrected version of Fig. 4, showing the correct data for the 48 h Control group in Fig. 4A, is shown below. It should be noted that the inadvertent error that occurred during the compilation of this figure did not affect the results or the conclusions of this article. The authors all agree to this Corrigendum, and are grateful to the Editor of *Oncology Reports* for granting them the opportunity to correct this figure. The authors also apologize to the readership for any inconvenience caused.

## Figures and Tables

**Figure 4.**
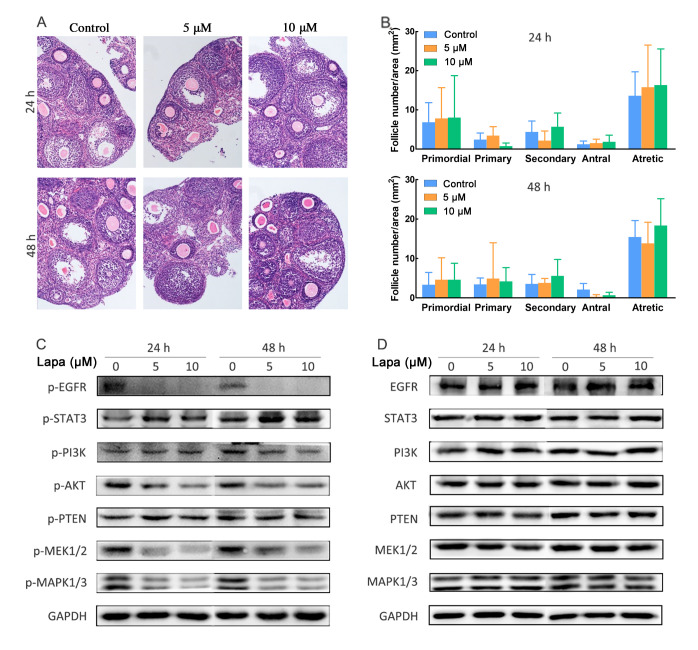
Histological assessment and western blot analysis of the ovarian slices *in vitro*. (A) Hematoxylin and eosin staining of ovarian slices cultured with or without lapatinib (5 and 10 µM) for 24 and 48 h (magnification, ×100). (B) Follicle numbers per area (mm^2^) of ovarian slices treated for 24 and 48 h. (C and D) Lapatinib (Lapa) inhibits the phosphorylation of EGFR (p-EGFR) and the PI3K/AKT and MAPK/ERK pathways; this inhibition was counteracted by activation of STAT3. EGFR, epidermal growth factor receptor; PI3K, phosphatidylinositol-3 kinase; AKT, protein kinase B; MAPK, mitogen-activated protein kinases; ERK, extracellular regulated kinase; STAT, signal transducers and activators of transcription; PTEN, phosphatase and tensin homologue deleted on chromosome ten.

